# Mercury in southern legal Amazonia: evaluating *Prochilodus nigricans* (Agassiz, 1829) as a bioindicator species and the risk of its consumption

**DOI:** 10.1007/s10661-026-15477-w

**Published:** 2026-05-19

**Authors:** Augusto Cesar da Costa Castilho, Kleber Solera, Vinícius José Santos Lopes, Gleyce Alves Machado, Ricardo Lopes Tortorela de Andrade, Leandro Dênis Battirola

**Affiliations:** 1 Federal University of Mato Grosso, Campus Cuiabá, Av. Fernando Corrêa da Costa, 2367, Boa Esperança, Cuiabá, MT 78060900 Brazil; 2 Federal University of Mato Grosso, Campus Sinop, Av. Alexandre Ferronato, 1200, Setor Industrial, Sinop, MT 78550000 Brazil; 3 3Federal University of Catalão, Setor Universitário, Avenida Dr. Lamartine Pinto Avelar, 1120, Catalão, GO 75704020, Caixa-Postal 56 Brazil

**Keywords:** Heavy metal, Artisanal and small-scale gold mining (ASGM), Public health, Environmental monitoring

## Abstract

Mercury (Hg), a highly toxic metal, continues to be widely used in artisanal and small-scale gold mining (ASGM), particularly in the Amazon region. Its improper use and disposal contribute to the contamination of aquatic ecosystems, impacting fish and the human populations that rely on them for food. This study evaluated mercury contamination, measuring total mercury (THg) in the Peixoto de Azevedo River Basin, located in the southern Brazilian Legal Amazon, by analyzing scale, skin, muscle, and liver tissues of *Prochilodus nigricans*, a key species in the local diet. Mercury was detected in all tissues and across all river stretches, with higher levels observed near mining sites, suggesting the association between ASGM and environmental contamination. The liver showed the highest THg levels (1.581 mg kg⁻^1^), followed by scales (0.537 mg kg⁻^1^), skin (0.153 mg kg⁻^1^), and muscle (0.087 mg kg⁻^1^). The results obtained for scales indicate their potential as a bioindicator matrix, allowing the use of less invasive sampling approaches for the species. Although muscle THg levels were below the FAO legal threshold (0.5 mg kg⁻^1^), the estimated health risk (THQ > 1) suggests potential harm to riverine populations due to high fish consumption. These findings underscore the urgent need for continuous monitoring and effective mitigation strategies to protect both aquatic ecosystems and public health in ASGM-affected regions.

**Supplementary Information:**

The online version contains supplementary material available at 10.1007/s10661-026-15477-w.

## Introduction

Species of *Prochilodus* (Agassiz, 1829) (Prochilodontidae, Characiformes) occur in the main hydrographic basins of South America, and they are characterized by high abundance and strong commercial appeal, being widely consumed and valued as a food source by local communities (Barros et al., 2021; Bayley et al., 2018; Pretto et al., 2020). *Prochilodus nigricans* (Agassiz, 1829), popularly known in Brazil as "curimba", "curimbatá" or "curimatã", is a migratory species and oligophagous detritivore that feeds on bottom sediments like organic debris, algae and microorganisms (Mota & Ruffino, 1997; Granado-Lorencio et al., 2005; Ohara et al., 2017; Bowen, 2021; Lacerda et al., 2024). It is found in white and black water rivers of the Amazon and Tocantins-Araguaia basins (Araújo-Lima & Ruffino, 2003; Santos et al., 2006). Because it is abundant, widely disseminated and fished in this region, *P. nigricans* is of great importance, both for subsistence and commercial fishing, to constitute one of the main species exploited in this region (Batista and Petrere, 2003; Barthem & Goulding, 2007; Batista et al., 2012; Monteiro-Filho et al., 2021).

However, the areas where *P. nigricans* occurs face increasing environmental pressures due to the exploitation of their natural resources, including the advance of the livestock and agricultural frontier, the expansion of cities, logging and mining. This is particularly true in the so-called "Arc of Deforestation", which includes the Southern Legal Amazon, in the mid-north of Mato Grosso (e. g. Fearnside, 2005; Silva et al., 2016; Silva et al., 2021; Teixeira et al., 2025; Rodrigues et al., 2013, 2016, 2021; Battirola et al., 2015).

In these areas, one of the most worrying impacts in relation to the conservation of natural ecosystems and their biodiversity is the dispersion and environmental contamination by toxic waste, especially those containing heavy metals (Casagrande et al., 2018, 2020; de Freitas et al., 2024; Solera et al., 2024). Among these metals are, for example, lead, mercury, cadmium, and chromium (Bakker et al., 2021), which, despite being natural elements, anthropogenic emissions raise their levels to the point of becoming toxic to the health of ecosystems and human health (Martoredjo et al., 2024; WHO, 2024). In the southern Amazon, because of illegal artisanal gold mining activities, mercury has been considered a genuine problem (Casagrande et al., 2023).

In fish, in general, the absorption of mercury occurs mainly through food, skin or scale and gills, generating growth problems, in their organs and in reproduction (Gomes & Sato, 2011). In this context, the hydrological cycle and changes in the diet of fish influence the concentrations of mercury absorbed or adsorbed by the species, which can bioaccumulate in distinct types of tissues (Paiva et al., 2022).

In general, several studies have demonstrated differences in mercury accumulation across tissues, with higher concentrations observed in the liver under heavy contamination, and in muscles under light contamination. The explanation given for this is that the mercury present in muscle tissue is bound to proteins rich in the amino acids cysteine ​​and methionine, which contain sulfur in their composition, and therefore exhibit a slower response to changes in mercury concentrations in the environment, reflecting the accumulation resulting from long-term exposure and predominantly associated with organic mercury. In contrast, liver tissue, due to its metabolic activity, reflects recent contamination, already manifested in a short period of exposure and being predominantly inorganic mercury (e.g. Havelková et al., 2008; Xu & Wang, 2015; Arantes et al., 2016a, 2016b; Teunen et al., 2022).

Although muscle tissue constitutes the primary route of human exposure to mercury, comprehensive environmental risk assessments require monitoring this metal across multiple fish tissues and, whenever possible, within the surrounding aquatic environment, to better elucidate contamination dynamics and ecological risks (e. g. Lima et al., 2002; Yi & Zang, 2012; Lima et al., 2015; Gomes et al., 2018; Kumar et al., 2023; Moraes et al., 2023).

Fish is a significant part of the population's diet in Latin American and Caribbean, and it is essential to assess the risk of mercury intake via this food source (Vergara et al., 2024). Studies carried out in the Amazon have shown that predatory fish may have higher concentrations of mercury in their biomass compared to species of lower trophic levels, this is due to mercury’s biomagnification capacity (e. g. Kasper et al., 2012; Lima et al., 2015; Arantes et al., 2016a, 2016b; Azevedo-Silva et al., 2016; Monteiro-Filho et al., 2021; Costa et al., 2022). A review conducted by Saidon et al., (2024) showed the mercury, because it is not metabolically degraded, results in a continuous increase in its concentration as it moves along the aquatic food chain, demonstrating that it can biomagnify from lower trophic levels such as particulate organic matter to higher trophic levels of fish.

In the southern Amazon, mercury (Hg) has been detected in different strata of native forest areas, with an average of 129.26 µg kg^−1^ for the leaf litter; 107.68 µg kg^−1^ for underwood; 99.08 µg kg^−1^ for herbaceous; 91.83 µg kg^−1^ for soil and 85.97 µg kg^−1^ for arboreal (Casagrande et al., 2023), and anthropized areas, where those closest to Artisanal and Small-scale Gold Mining (ASGM) have the highest contamination with values of 53.0 µg kg^−1^ in *Gycine max* (Casagrande et al., 2020); 172.8 µg kg^−1^ in *Moquilea tomentosa* (Deecken et al., 2022) and 92.35 µg.kg^−1^ in the root of *Manihot esculenta* (Malvino et al., 2023), impacting fish species such as *Hydrolycus armatus* (Jardine and Shomburgk, 1841) (Cynodontidae) with 0.229 mg kg^−1^; *Boulengerella cuvieri* (Agassiz, 1829) (Ctenoluciidae) 0.199 mg kg^−1^ and *Serrasalmus rhombeus* (Linnaeus, 1766) (Serrasalmidae) 0.304 mg kg^−1^ (Matos et al., 2021). In light of the growing global concern regarding mercury contamination in water bodies caused by anthropogenic activities, and its subsequent impact on human populations that consume fish from these environments (Basta et al., 2023; Lopes et al., 2016), it is essential to assess mercury levels in species consumed by local communities.

A literature review conducted by Sousa et al., (2024) found that most non-predatory Amazonian fish are within established limits, although three non-predatory species exceeded the safety limit for human consumption: *Curimata* sp. in the Tapajós River, *Hypophthalmus edentates* in the Purus River, and *Hypophthalmus marinatus* in the Negro River.

A study by Matos et al., (2025) in the Teles Pires River showed that *P*. *nigricans* had the lowest mercury concentrations in its muscle but the highest concentrations in its liver when compared to piscivorous species. According to the authors, this situation is related to the fact that detritivores feed on sediments, a site of high mercury deposition. Its broad distribution, ease of sampling and identification (Loureiro et al., 2023), combined with evidence of mercury accumulation across all analyzed tissues, support its suitability as a bioindicator of mercury presence, particularly in benthic environments. In addition to being an important food and economic resource in the region, *P. nigricans*, playing a relevant role in the structuring of trophic networks and nutrient cycling, and showing strong potential for use in environmental biomonitoring studies.

Additionally, this study examined the suitability of *P. nigricans* as a bioindicator of mercury contamination in the Peixoto de Azevedo River basin, in the southern Brazilian Legal Amazon. Specifically, this study sought to answer the following questions: (i) does *P. nigricans* accumulate mercury in a tissue-specific manner in this river basin? (ii) can less invasive tissues, such as scales and skin, be used as alternative matrices for environmental mercury biomonitoring? and (iii) do mercury levels in muscle tissue indicate potential risks to human health under regional fish consumption scenarios? Addressing these questions provides support for improving mercury monitoring approaches and informing public strategies aimed at mitigating contamination in regions where this species is widely distributed.

## Material and methods

### Study area

**Fig. 1:**
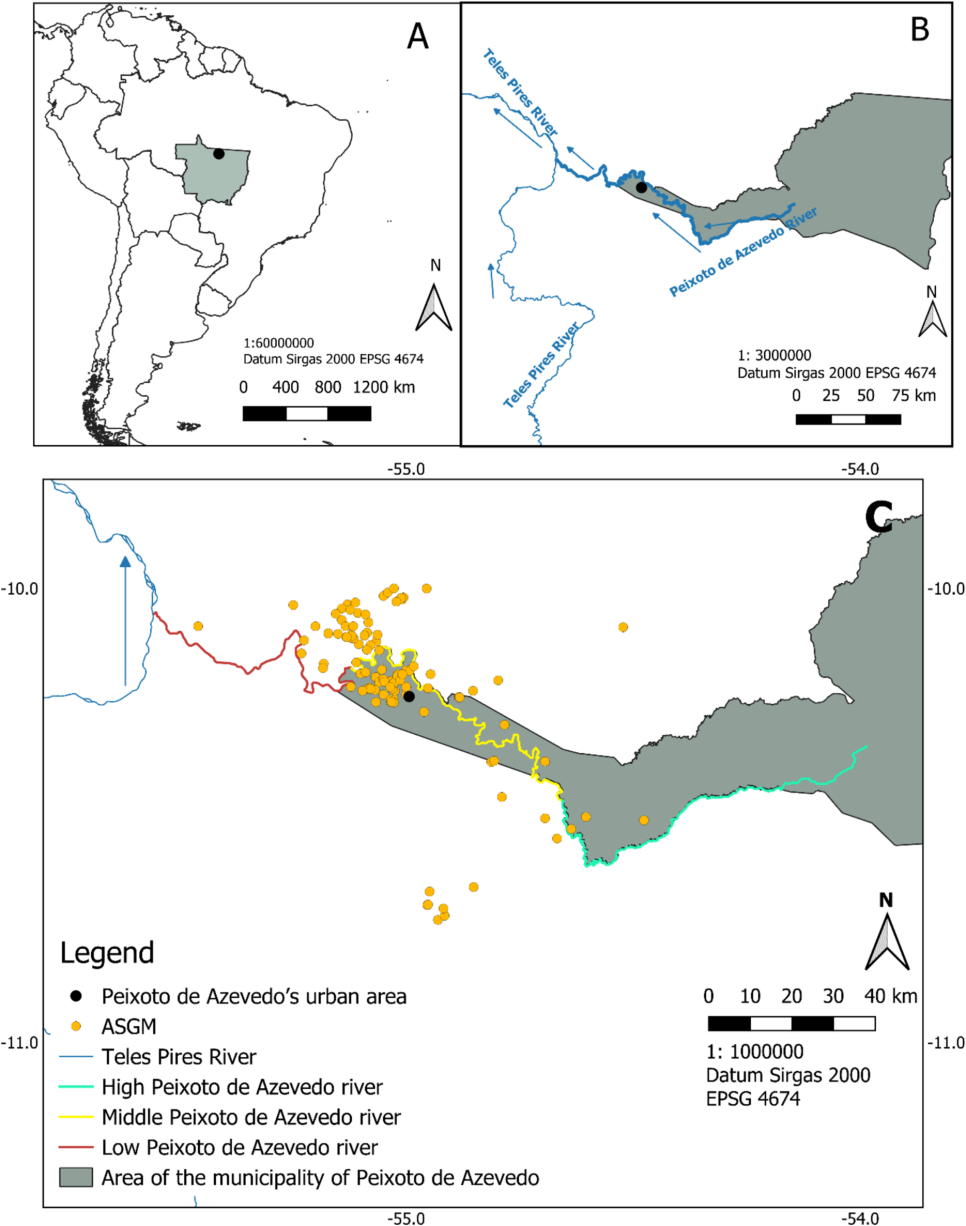
Study area: **A** location in South America, highlighting Mato Grosso; **B** Peixoto de Azevedo municipality, urban area, and Peixoto de Azevedo river; **C** fishing zones (upper, middle and lower Peixoto de Azevedo river) and ASGM sites. The arrows indicate the direction of the river, and the location of ASGM is public information from the Brazilian government’s National Mining Agency (ANM)

This study was developed in the northern region of Mato Grosso, south of the Brazilian Legal Amazon, specifically, in the basin of the Peixoto de Azevedo River (Fig. 1). The river is a tributary of the right bank of the Teles Pires River, one of the sources of the Tapajós River. The Peixoto de Azevedo river basin has a perimeter of 754 km, an area of 19.608 km^2^ and a length of 330 km. The basins are in the northern region of Mato Grosso (Marcelino et al., 2019). In this river, as in others in the Amazon region, gold prospecting occurs through random drilling to verify the presence of gold-bearing gravels, which can lead to unnecessary removal of the forest and remobilization of sediments from the riverbed, thus intensifying silting and erosion processes (Seimetz et al., 2020). Such mining activities had a great boost from 1979, when gold was found in abundance in region (Ferreira & Silva, 2008). Furthermore, this river exhibits significant horizontal spreading during its flood season, carrying a large amount of sediment from its banks into the riverbed, giving it the sedimentary characteristics of a white-water river.

The climate is monsoon tropical, according to the Koppen classification system (Alvares et al., 2013). The region has an altitude of 300–400 m and an average temperature between 24.3 and 24.8ºC. Annual rainfall varies between 2,000 and 2,300 mm (Corsini, 2018). The region is part of the North Mineral Province, which has gold and other base metals in the Peixoto de Azevedo-Teles Pires-Aripuanã Gold Province (Lacerda Filho et al., 2004). The Peixoto de Azevedo Mining Reserve is one of the largest gold extraction areas in Brazil, standing for approximately 4.0% of national production (Brasil, 2024).

The course of the Peixoto de Azevedo River is divided into upper, middle, and lower sections, as previously characterized by Casagrande et al., (2023) (Fig. 1). The upper Peixoto de Azevedo corresponds to the area closest to the source of the river, therefore, further away from the region of focus of gold mineral exploration. The relief of this area is higher than in other regions, and it has more nearby native forests and lower incidence of mineral exploration.

The area of the middle Peixoto de Azevedo, in turn, encompasses the central region of the river, being the closest to the urban area of the municipality of Peixoto de Azevedo. It has gold exploration points and is considered the region with the highest rate of mercury emissions due to mining, which is why it is also a region with few areas of preserved native forest. The lower Peixoto de Azevedo is the region that encompasses the mouth of the river, near its meeting with the Teles Pires River. The relief is moderately lower than the other areas. Mining is an intermediary between the other areas. In relation to vegetation, it has few areas of primary vegetation (Casagrande et al., 2023).

### Procedures in the field

**Fig. 2:**
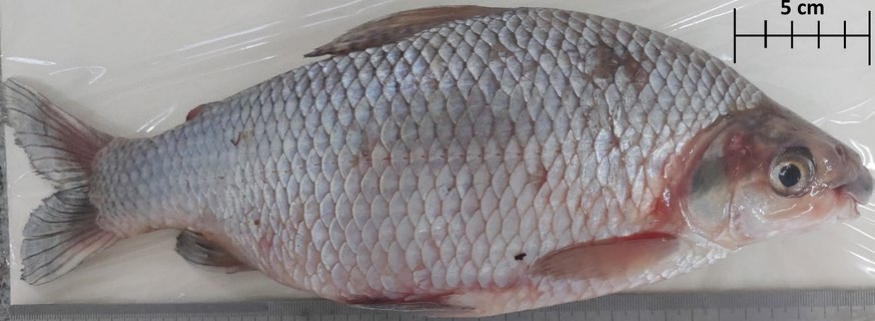
Specimen of *Prochilodus nigricans* obtained from the Peixoto de Azevedo River, southern Brazilian Amazon

For the analysis of mercury concentration in different tissues (scale, skin, muscle, and liver) of *P. nigricans* (Fig. 2), the specimens were purchased from professional fishermen linked to cooperatives, caught between the months of July and August 2023, the dry season in the region. In this period, the Peixoto de Azevedo River presents itself with its main channel well defined, but with points of difficult navigation. It is important to highlight that the fishers of these cooperatives have restricted fishing areas, they never go beyond their fishing grounds; thus, it was possible to guarantee the separation by origin of the analyzed specimen, separating them according to the three regions of the Peixoto de Azevedo River, being the upper, middle and lower (Fig. 1).

Because of the dry period, there were difficulties in navigating the river to obtain specimens, which led to variations in the quantity sampled depending on the site, with nine specimens obtained from the upper Peixoto River, 10 from the middle Peixoto River and 14 from the lower Peixoto River, totaling 33 individuals analyzed, with 27.1 ± 5.9 cm and 477.8 ± 259.7 g. All specimens were kept frozen after fishing, for transport to the laboratory. The species identification was confirmed using the Ichthyological Collection of the Southern Amazon Biological Archive (ABAM-UFMT).

### Laboratory procedures

#### Sample preparation

All laboratory procedures were performed at the Trace Metals Laboratory of the Federal University of Mato Grosso, campus Sinop (UFMT), where the 33 specimens were thawed and washed with water only, allowing the analysis of the scales and skin to measure both adsorbed and absorbed mercury, with the aid of stainless-steel surgical instruments properly sanitized, samples were taken from the liver, the dorsolateral muscle of the region above the lateral line, the skin, and the scale. However, during the sample removal procedures, only 29 livers were analyzed, and the others were lost due to their fragility during the handling of the specimen.

After the removal of the tissue samples, the material was dry in an air circulation oven at a temperature of 50 °C, up to constant weight. The samples, after drying, were mechanically crushed with the aid of a knife mill, sieved through a 2 mm sieve for homogenization, and stored individually in properly identified paper bags in a freezer at −20ºC until the determination of the total mercury THg concentration.

#### Mercury analysis

For the total mercury chemistry analysis, 0.3 g of each sample was weighed on a digital scale with an accuracy of ± 0.1 mg in Shimadzu ATX 224. Subsequently, the samples were placed in digestion tubes by adding 2.0 mL of nitric acid and perchloric acid solution in the proportion of 1:1 and 5.0 mL of analytical grade sulfuric acid (H_2_SO_4_). The tubes were heated in a digester block at 230 °C for 30 min (Akagi & Nishimura, 1991). Then, after cooling at room temperature, the extracts were transferred to volumetric flasks (25 mL), completing the contents with distilled water.

The total mercury (THg) content was determined in Agilent AA240FS atomic absorption spectrometry equipment, coupled with a VGA77 vapor generation accessory, using the methodology of Akagi and Nishimura (1991). The standard stock solution used for the calibration curve is traceable to NIST (*National Institute of Standards and Technology*), Specsol® brand. For every 10 samples analyzed, one replicate was made to control the accuracy of the total mercury measurements. Replicates not performed with liver samples due to the low mass of the material. In all, 139 samples with 11 replicates were analyzed, the coefficient of variation between these replicates was the same as that found in the samples 3.73%.

To evaluate the accuracy and precision of the analysis, we used ERM-BB422 certified reference sample of fish muscle, sample 588, produced by the Institute of Reference Materials and Measurements of the Joint Research Centre of the European Commission, which was certified with 601 ± 30 μg kg^−1^ of Hg. In our laboratory, the average value recovered was 644 ± 24 μg kg^−1^ (107% of the certified value), with a coefficient of variation of 3.73%, proving the quality of the chemical analysis.

The detection limit, defined as the mean of ten blanks plus three times the standard deviation, was 0.010 mg kg^−1^, while the limit of quantification, defined as the mean of ten blanks plus 10 times the standard deviation, was 0.0149 mg kg^−1^.

To comply with the legislative standards, the entire analysis was carried out by means of wet weight, so that the concentration obtained was converted by the factor corresponding to the moisture content lost, with this, considering that each evaluated part presented a different loss, we obtained the following conversion factors: scale 1.860 (46.2%); skin 3.058 (67.3%); muscle 4.325 (76.9%) and liver 3.813 (73.8%). These factors were obtained by dividing the sum of wet weight by the sum of dry weight (Ʃ wet weight/Ʃ dry weight) and the factor was calculated for each of the tissues analyzed.

### Data analysis

The results obtained were transformed using a base 10 logarithm and underwent the D’Agostino-Pearson normality test and after verifying normality, analysis of variance (ANOVA) was performed. The results of the ANOVA were, submitted to an evaluation of means using the Scott-Knott test at 5%, to compare the variable THg concentration between the parts of the fish and the place of collection (Local group with variables, upper Peixoto River, middle Peixoto River and lower Peixoto River; Part group with variables, scale, skin, muscle and liver). The analyses were conducted using the R Program version 4.4.2 of 09/01/2024, the R package used was the tidyverse (ggplot2; dplyr) obtained directly from CRAN (Comprehensive R Archive Network).

Considering that, the health damage caused by mercury toxicity is directly related to its intake, and to ascertain the food safety regarding the consumption of *P. nigricans* by the population, the estimates of weekly and daily intake (EWI and EDI, respectively) and the target risk quotient (THQ) were calculated. The concentration of mercury used for the calculation was the average found in the muscle of *P. nigricans*. The mean age for analysis was 70 years, and the mean body mass was 70 kg.

There are no studies on the per capita fish consumption of the population of Peixoto de Azevedo, Mato Grosso, Brazil. Therefore, consumption data from Isaac and Almeida (2011) were considered, encompassing both the general Amazonian riverine population and that of the Mato Grosso Amazon (representing the nearest context to the riverside communities of the Peixoto de Azevedo). Additionally, data from Costa et al., (2025) were included, referring to the population of the lower Teles Pires River, which, although not exclusively riverine, is geographically closest to the study area. Lastly, estimates reported by Oliveira et al., (2021) for Tupari ethnic group of the Rio Branco Indigenous Reserve. Accordingly, the calculations carried out using the equations according to FAO/WHO. (2017) as applied by Porto et al., (2024):

$$EWI=\frac{CHg . IR}{BW}$$

$$THQ=\frac{IR . ED . EF . {C}_{Hg}}{BW . AT . RD} . {10}^{-3}$$

$$MSCQ=\frac{BW . RD}{{C}_{Hg}} . {10}^{3}$$

where:

EWI: estimated weekly intake.
CHg: mercury concentration in fish muscle (0.087 mg kg^−1^).
IR: feed intake rate in terms of mass in grams per week.
THQ: Target hazard quotient.
ED: exposure duration.
EF: exposure frequency (365 days per year).
BW: body weight (70 kg).
AT: average time of consumption of the food (EF. 70 years).
RD: oral reference dose, (which is 0.0003 mg kg^−1^ per day for mercury).
MSCQ: maximum safe consumption quantity.

## Results

**Fig. 3:**
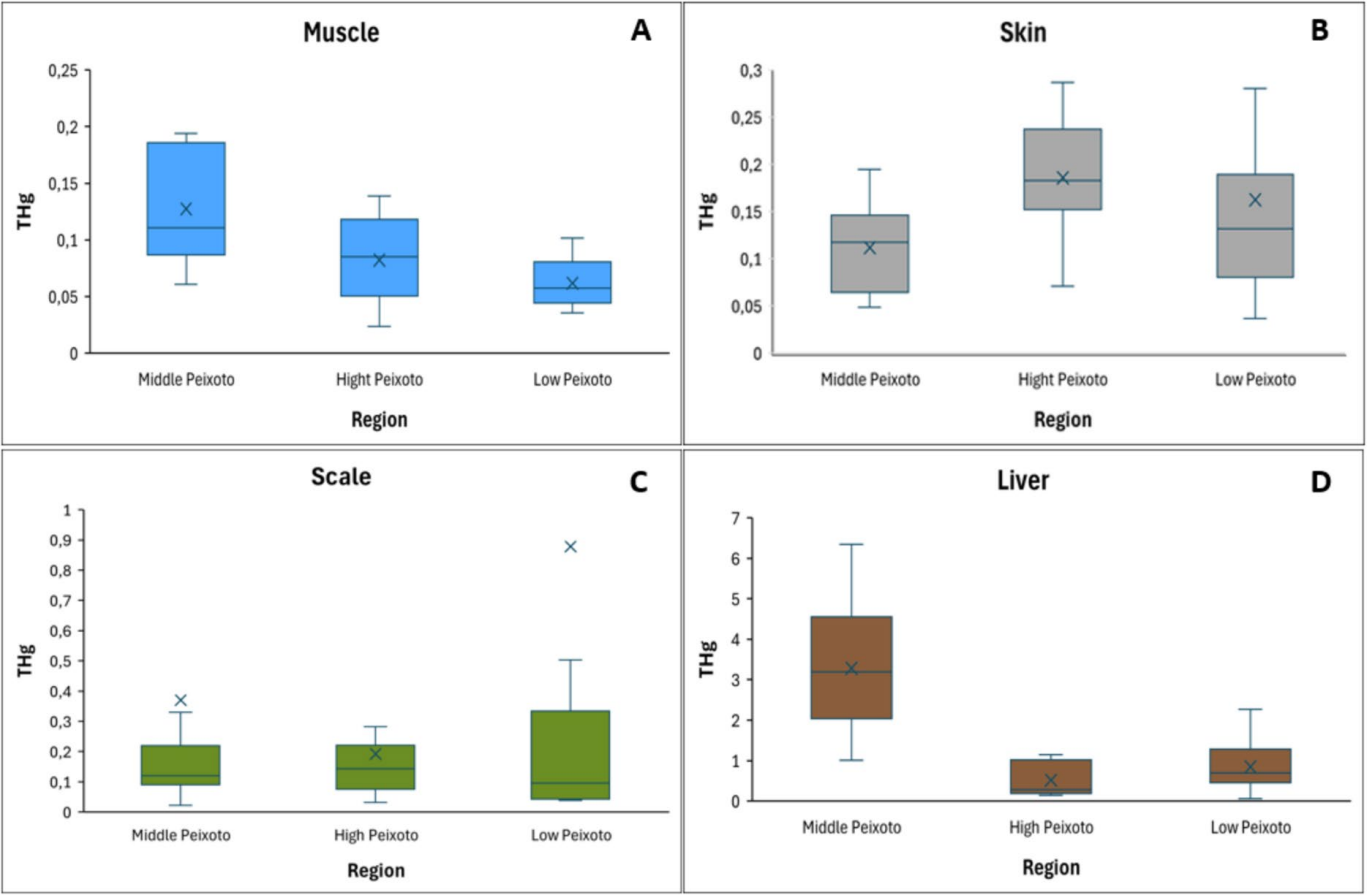
Distribution of THg (mg kg.^−1^) content within different tissue (scale, muscle, skin and liver) of *Prochilodus nigricans*, collected in different regions of the Peixoto de Azevedo River (middle, high and low)

Mercury was detected in all samples and tissues of *P. nigricans*. Significant variation in mean mercury concentrations was observed among the regions of the Peixoto de Azevedo River (F = 7.2, p < 0.05), with the highest values recorded in the middle region (0.972 ± 1.604 mg kg⁻^1^), while the upper (0.235 ± 0.277 mg kg⁻^1^) and lower (0.459 ± 1.449 mg kg⁻^1^) regions showed no significant differences. Among tissues (F = 48.7, p < 0.05), the liver presented the highest mean concentration (1.58 ± 1.621 mg kg⁻^1^), followed by scales (0.537 ± 1.803 mg kg⁻^1^), skin (0.153 ± 0.100 mg kg⁻^1^), and muscle (0.089 ± 0.043 mg kg⁻^1^), with a marked difference between muscle and liver (ESM 1). Spatial patterns varied by tissue: muscle and liver showed maximum concentrations in the middle region, whereas skin peaked in the upper region and scales in the lower region. Two outlier values for scales were removed to improve graph visualization, although mean and median indicators were retained (Fig. 3).

Although the highest average value was in the middle Peixoto de Azevedo River, followed by the lower Peixoto de Azevedo River and the upper Peixoto de Azevedo River, respectively, this pattern does not hold when analyzing the different tissues. Muscle and liver showed higher concentrations of mercury in the middle Peixoto River, while scale had higher averages in the lower Peixoto de Azevedo River, and skin in the upper Peixoto de Azevedo River (F = 3.9, p < 0.05) (Fig. 3).

As for the risk of contamination of the human population associated with fish consumption, the World Health Organization (WHO) considers that the tolerable weekly intake (PTWI) is 1.6 μg kg^−1^ of THg. For this purpose, the target hazard quotient (THQ) is used, where THQ < 1 means safe consumption, while THQ ≥ 1 represents a potential risk to health (WHO, 2007; EFSA, 2012).

The data collected show that the largest safe amount of *P. nigricans* from the Peixoto de Azevedo River is 241.379 g/day, having generated a THQ value of 0.485. Thus, in view of the average amount of fish consumed by the riverine population in the Mato Grosso Amazon (117.014 g/day, THQ = 0.485), there is no restriction on their consumption. The data reveal that for an average consumption of the riverine population of the Brazilian Amazon (259.397 g/day) the *P. nigricans* obtained from the Peixoto de Azevedo River in Mato Grosso is a health risk, but not considering the average consumption of the population in Mato Grosso. Only if the average fish consumption were the same as that of the riverine population of the Brazilian Amazon as a whole (259.397 g/day) would it be necessary to impose any restrictions (Table 1).

**Table 1:** Estimated mercury intake through fish consumption via estimated weekly intake (EWI), estimate daily intake (EDI) and target hazard quotient (THQ), based on mercury concentration obtained in *Prochilodus nigricans* tissues in the Peixoto de Azevedo River, southern Amazonia, Brazil-MT

Local	Consumption (g/day)	EWI	EDI	THQ
Riverine population of the Brazilian Amazon (Isaac & Almeida, 2011)	259.397	2.257	0.322	1.075
Riverine population of the Mato Grosso Amazon (Isaac & Almeida, 2011)	117.014	1.018	0.145	0.485
Indigenous Land Population in Rondônia (Oliveira et al., 2021)	105.556	0.918	0.131	0.437
Population under Teles Pires in Mato Grosso (Costa et al., 2025)	15.123	0.132	0.019	0.063

## Discussion

Mercury reaches aquatic sediments through multiple pathways, including direct discharge from riverbank and bed mining by dredges or rafts, and organic matter input via bank flooding or runoff that carries litter to the main channel. In forested areas, litter acts as a mercury sink, contributing to its soil deposition (Casagrande et al., 2023).

The species *P. nigricans* is an oligophagous detritivore that feeds on bottom sediments (Lacerda et al., 2024), with a diet based on benthic biofilm, a flocculent matrix of algae, bacteria, and dead organic matter, (Bowen, 2021). Its broad distribution, ease of sampling and identification (Loureiro et al., 2023), combined with evidence of mercury accumulation across all analyzed tissues, support its suitability as a bioindicator of mercury presence, particularly in benthic environments.

Gills, liver, and kidneys are the organs most susceptible to accumulation, with different responses depending on the metal, constituting important tools in bioindication these elements in the environment (Shah et al., 2020). The results presented for the Peixoto de Azevedo River basin, southern Amazonia, show the presence of mercury, with variable concentrations as a function of the different regions of the basin and the tissues of *P. nigricans*, having presented the liver as the tissue with the greatest accumulation and thus demonstrating its susceptibility in relation to the other tissues analyzed.

About the different tissues evaluated, it was seen that the scales presented a higher concentration of mercury in relation to the muscle, regardless of the fishing location. This concentration of mercury in the scale shows the possibility of using them as a non-lethal and less invasive sampling middle for analyzing mercury in fish, another advantage of using scales is their ease of storage for analysis, as they do not degrade as easily as muscle or liver. Furthermore, the scale will contain both absorbed and adsorbed mercury, providing a more accurate diagnosis of the environmental situation, however, further studies with mercury on fish scales are recommended to obtain an acceptable protocol for its use in monitoring.

Cervenka et al., (2011), except for species with tiny scales, have already demonstrated the use of scales to measure mercury content in fish. Other studies say that scale can be used as sorption material to remove pollutants from aquatic environments (e. g. Bazarin et al., 2019; Santos et al., 2009; Shaikhiev et al., 2020; Zayadi & Othman, 2013). However, the use of scales is only for environmental monitoring and cannot replace muscle measurement when analyzing the risk to population due to fish consumption.

Prochilodontids take advantage of food resources from detritus (Silva & Stewart, 2017), so that, when turning over the sediment in search of food, both the contaminated organic material and the sedimented metallic mercury can be adsorbed to the scale. It is important to note that *P. nigricans* have large overlapping scale, which helps the accumulation of this material. Species with smaller scales and that do not forge in the bottom sediment may not have the same potential for this type of monitoring, since the larger the available area, the greater the possibility of mercury adsorption.

Results show any of the analyzed tissues can be used to monitor mercury in the environment, and edible parts should not be ignored for analysis regarding contamination due to consumption. The same can be said in relation to the skin, in which relevant concentrations of mercury were recorded in the upper and lower Peixoto de Azevedo River, higher than obtained on muscle.

Obviously, when it comes to food safety risks, mercury in muscle tissue should always be measured. In this sense, the results obtained reinforce that the concentration of mercury is higher in the muscle of fish near areas with a high concentration of ASGM (0.128 mg kg^−1^) and lower in areas with a lower concentration of ASGM (0.062 mg kg^−1^). Similar values were found in muscle or *P. nigricans* in the Teles Pires river (0.05 mg kg^−1^) reaching up to 0.198 mg kg^−1^ in fish obtained from the Peixoto de Azevedo river (Matos et al., 2025), these authors also reported values between 0.04 mg kg^−1^ and 0.09 mg kg^−1^ in muscle of *P. nigricans* to Amazon River basin; 0.05 mg kg^−1^ in the Solimoes River, and 0.024 mg kg^−1^ for the Madeira River.

Regarding the current legislation, the Brazilian standard postulated by Normative Instruction No. 160 of the National Health Surveillance Agency (ANVISA), published in the Federal Official Gazette No. 126 of June 6, 2022 (Brasil, 2022), establishes that the maximum tolerated limit of mercury in predatory fish is 1.00 mg kg^−1^, in wet weight, while for non-predatory fish this limit is 0.50 mg kg^−1^. These values are consistent with the UN General Standard for Contaminants and Toxins in Food for Human Consumption (FAO, 1995). The FAO recommends that the consumption of fish with 0.5 mg kg^−1^ mercury should not exceed 192 g per person per week and may reach 960 g per person, per week, in the case of fish with up to 0.1 mg kg^−1^ mercury (FAO/WHO., 2017).

In the Peixoto de Azevedo River, the average mercury in the muscle of *P. nigricans* was 0.087 mg kg^−1^ and the calculated risk of consumption occurs for amounts above 241.379 g/day, or 1,689.65 g/week. Although the values obtained indicate food safety according to both Brazilian standards and FAO recommendations, it is important to note that the average fish consumption of the riverine population in the Brazilian Amazon (259.397 g/day) exceeds these limits. This represents a potential health risk, and therefore, the consumption of *P. nigricans* from the Peixoto de Azevedo River should be restricted for this population group, especially for children and pregnant women, considering the population’s susceptibility to problems caused by mercury, particularly those involving tissue formation and the central nervous system (e.g. Dack et al., 2022).

It should not forget that, due to biomagnification, predatory fish will have a higher mercury content than detritivores fish, and constant monitoring of the Peixoto de Azevedo River should be carried out with predatory species to ensure safety in the consumption of their fish. Such monitoring is also justified by the fact that Brazil is among the countries with fish with the highest levels of mercury, constituting a greater health risk, as it has more than 1/3 of the species with THQ ≥ 1 (Vergara et al., 2024).

Our study, along with others conducted on *Prochilodus* species*,* the concentrations in muscle tissue remained below the limit established by the FAO (FAO, 1995) and determined by different control agencies, such as ANVISA in Brazil (Brasil, 2022), the European EFSA (European Commission, 2023), the FDA of the United States (FDA, 2022), or the Japanese MHLW (JETRO, 2010).

Among the species evaluated, we can mention *Prochilodus lineatus* (Vaenciennes, 1836) in the Pantanal of Mato Grosso (Santos Filho et al., 2016), *Prochilodus* cf *beni*, *P. nigricans*, *Prochilodus theraponura* (Fowler, 1906) in the Madeira River, Brazilian Amazon (Bastos et al., 2008) and *Prochilodus mariae* (Eigenmann, 1922) in the Colombian Amazon Valbuena-Rodríguez & Navarro-Ramírez, 2021). However, mercury levels, above the maximum tolerance limit, have already been recorded for *Prochilodus magdalenae* (Steindachner, 1879) in the Colombian Amazon (Cruz-Esquivel & Marrugo-Negrete, 2022; Salazar-Camacho et al., 2020).

Liver tissue tends to have higher concentrations of Hg than muscle tissue due to its metabolic properties, ability to induce metallothionein synthesis, and its functions in the storage, redistribution, detoxification, and transformation of xenobiotics (Jesus et al., 2011; Monteiro et al., 2013; Silva & Lima, 2020). Unlike muscle tissue, this tissue is more sensitive to short-term changes, being able to accumulate higher concentrations of mercury compared to the others (Rua-Ibarz et al., 2019).

Changes in mercury exposure are first reflected in liver tissue concentrations, and only later in other organs and tissues (Xu & Wang, 2015). As it is a detoxification organ, the presence of mercury shows recent exposure to the contaminant. On the contrary, muscle tissue tends to function as a long-term reservoir of Hg, with the function of protecting other organs. Thus, the mercury present in the muscle tissue of fish is the legacy mercury, that is, it stands for a late exposure to the pollutant (Vieira et al., 2021). These characteristics show that the high mercury content found in the liver, with an average of 1.581 mg kg^−1^ in the Peixoto de Azevedo River, is associated with recent contact with the metal, as well as its presence in all regions of the river.

Similarly to what was seen for *P. nigricans*, Gomes et al., (2021) obtained higher concentrations of mercury in the liver of *Prochilodus costatus* (Valenciennes, 1850) and *Prochilodus argenteus* (Agassiz, 1829), when compared to muscle tissue in the São Francisco River basin. This pattern seems to be recurrent, being recorded for other Amazonian species such as *Plagioscion squamosissimus* (Heckel, 1840) (Sciaenidae) in the Machado River in Rondônia (Costa et al., 2022), and for *Brycon falcatus* (Müller and Troschel, 1844) (Characidae) in the Teles Pires River in Mato Grosso (Matos et al., 2018)*.* According to da Silva et al., (2022), in a study with *P. argenteus*, hepatic tissue was identified as the most appropriate bioindication matrix for mercury, attributed to the low binding affinity of mercury to muscle proteins, which explains the pattern found.

In general, it was found that the presence of mercury is not uniformly distributed in the Peixoto de Azevedo river basin. The middle Peixoto de Azevedo River, which corresponds to the area with the highest concentration of mining activities, was also where the highest average mercury concentration values were obtained for the muscle tissues, (0.128 mg kg^−1^), as recorded in the studies by Casagrande et al., (2020, 2023); Salazar-Camacho et al., (2021); Malvino et al., (2023); and Eckley et al., (2023). Since muscle tissue is the edible part, the result warns that consumption of this fish forms this region should not exceed 960 g per week, as recommended by FAO (1995).

It is understood that the high concentration of Artisanal and Small-Scale Gold Mining in this region is associated with the highest concentrations of mercury recorded. Rodrigues et al., (2022) showed that the concentration of mercury in the soil stays high up to 100 m away from the source, and can be reached for up to 1 km, and can reach water bodies. We highlight that a large part of artisanal gold mining in this region takes place in the riverbed itself or on the banks of rivers, contributing greatly to the contribution of suspended material and directly mercury, in addition to generating other severe environmental impacts (Gasparinnetti et al., 2024).

During the flood period of lowland rivers such as the Peixoto de Azevedo River, when overflowing and flooding the riparian forests, they can lead to contamination in the forest area, as well as bring the contamination that may exist in these places. The soil of flooded areas may have a concentration of mercury higher than the area of the river channel due, among other factors, to the accumulation of macrophytes deposited during the flood period (Pestana et al., 2019). This seasonal process of water level variation interferes with fish feeding and reproduction and, so, can interfere with the mercury content found in aquatic species Paiva et al., (2022).

## Conclusion

This study addressed three central questions regarding mercury contamination in the Peixoto de Azevedo River basin. First, *Prochilodus nigricans* exhibited clear tissue-specific patterns of mercury accumulation, with higher concentration observed in metabolically active tissues, confirming that mercury bioaccumulation in this species is not homogeneous among tissues. Second, the concentrations detected in less invasive matrices, particularly scales, support their feasibility as complementary tools for environmental mercury biomonitoring, offering practical advantages for long-term monitoring programs. Nevertheless, liver tissue remained the most sensitive matrix for detecting recent exposure.

Third, although mercury concentrations in muscle tissue were below the limits established by Brazilian legislation, the estimated consumption scenarios indicate that intake should be carefully managed, highlighting the importance of considering local dietary habits when assessing potential human health risks.

Overall, these findings support the use of *P. nigricans* as a bioindicator species within integrated mercury monitoring strategies in the southern Brazilian Legal Amazon. The results reinforce the need for continuous environmental surveillance, especially in areas subject to anthropogenic pressures, and provide relevant scientific support for public policies aimed at environmental protection, food safety, and public health in the region.

## Supplementary Information

Below is the link to the electronic supplementary material.

Supplementary file1 (PDF 118 KB)

## Data Availability

No datasets were generated or analysed during the current study.
