# Supplementary material for: Mercury in southern legal Amazonia: evaluating Prochilodus nigricans (Agassiz, 1829) as a bioindicator species and the risk of its consumption
Source: Environ Monit Assess. 2026 May 19;198(6):611. doi: 10.1007/s10661-026-15477-w (PMC13186798; doi:10.1007/s10661-026-15477-w)
Supplement: Supplementary file 1 — Supplementary file1 (PDF 118 KB) [file 10661_2026_15477_MOESM1_ESM.pdf]

ESM 1. Mean THg concentration (mg kg^{-1}) in different tissues of *Prochilodus nigricans*, from different regions of the Peixoto de Azevedo River, southern Amazonia, Brazil-MT.

Region/Tissue	Muscle	Skin	Scale	Liver	Average
**High**	0.082 aA ±0.027	0.186 aA ±0.063	0.193 aA ±0.204	0.513 aB ±0.419	0.235 a ±0.277
**Middle**	0.128 aA ±0.049	0.112 aA ±0.047	0.370 aA ±0.754	3.281 bB ±0.419	0.972 b ±1.604
**Low**	0.062 aA ±0.021	0.163 aB ±0.137	0.879 aB ±2.712	0.842 aC ±0.654	0.459 a ±1.449
**Average**	0.087 A ±0.043	0.153 B ±0.100	0.537 B ±1.803	1.581 C ±1.621	

Different lowercase letters show significant differences between the regions (High, Middle and Low) while different uppercase letters show a difference between the tissues, considering a probability of 5% by the Scott-Knott test.
